# HMGA1 and FOXM1 Cooperate to Promote G2/M Cell Cycle Progression in Cancer Cells

**DOI:** 10.3390/life13051225

**Published:** 2023-05-22

**Authors:** Qingfang Zheng, Ziyang Luo, Mingjun Xu, Shazhou Ye, Yuxin Lei, Yang Xi

**Affiliations:** Institute of Biochemistry and Molecular Biology, Basic Medical Sciences, Health Science Center, Ningbo University, Ningbo 315211, China; zhengqf910@163.com (Q.Z.); baronlzyang@163.com (Z.L.); 1911074035@nbu.edu.cn (M.X.); yeshazhou@foxmail.com (S.Y.); 216002284@nbu.edu.cn (Y.L.)

**Keywords:** HMGA1, FOXM1, cell cycle, cancer, cell proliferation

## Abstract

HMGA1 is a chromatin-binding protein and performs its biological function by remodeling chromatin structure or recruiting other transcription factors. However, the role of abnormally high level of HMGA1 in cancer cells and its regulatory mechanism still require further investigation. In this study, we performed a prognostic analysis and showed that high level of either HMGA1 or FOXM1 was associated with poor prognosis in various cancers based on the TCGA database. Furthermore, the expression pattern of HMGA1 and FOXM1 showed a significant strong positive correlation in most type of cancers, especially lung adenocarcinoma, pancreatic cancer and liver cancer. Further analysis of the biological effects of their high correlation in cancers suggested that cell cycle was the most significant related pathway commonly regulated by HMGA1 and FOXM1. After knockdown of HMGA1 and FOXM1 by specific siRNAs, an obvious increased G2/M phase was observed in the siHMGA1 and siFOXM1 groups compared to the siNC group. The expression levels of key G2/M phase regulatory genes PLK1 and CCNB1 were significantly downregulated. Importantly, HMGA1 and FOXM1 were identified to form a protein complex and co-located in the nucleus based on co-immunoprecipitation and immunofluorescence staining, respectively. Thus, our results provide the basic evidence that HMGA1 and FOXM1 cooperatively accelerate cell cycle progression by up-regulating PLK1 and CCNB1 to promote cancer cell proliferation.

## 1. Introduction

According to a 2019 evaluation by the World Health Organization (WHO), cancer is the top leading cause of death before age 70 in more than 60% of countries. Globally, approximately 18.1 million new cancer cases and nearly 10 million cancer deaths were reported in 2020, excluding non-melanoma skin cancers. By 2040, global cancer cases will reach 28.4 million, a projected 47% increase from 2020 [1]. Cancer cells are known to rely on high levels of transcription to survive and maintain their malignant phenotype [2]. When cancer cells are subjected to transcriptional stress, some genes are induced to be overexpressed and usually related to tumor growth, metastasis, and chemoradiotherapy resistance [3,4].

As a non-histone chromatin protein, the high-mobility group AT-Hook 1 (HMGA1), which is highly expressed in embryos and barely detectable in adult tissues, is repopulated in tumor cells [5,6,7]. HMGA1 remodels chromatin structure and participates in multiple fundamental cellular processes, including cell proliferation, differentiation, apoptosis, DNA repair, and cancer development [8,9,10]. Previous studies confirmed that HMGA1 can regulate the progression of cervical cancer through promoting G1/S phase transition by activating transcription of cyclin E and cyclin D [11]. However, ectopic expression of HMGA1 in hepatocellular carcinoma blocks the G0/G1 to S transition, indicating a cell-type-dependent function of HMGA1 [12].

Although HMGA1 protein lacks intrinsic transcriptional activity itself, it exerts biological functions by remodeling chromatin structure and recruiting other transcription factors to form higher-level transcriptional complexes or enhancer bodies [13,14]. Forkhead box M1 (FOXM1) interacts with a variety of signaling pathways, directly or indirectly activates the transcriptional expression of target genes and participates in a variety of physiological and pathological processes, including cell proliferation, angiogenesis, tumor transformation, invasion and metastasis [15,16]. Studies have shown that FOXM1 is a key interactor with HMGA1, and the binding of FOXM1 to the HMGA1 promoter activates HMGA1 gene transcription [17]. Meanwhile, FOXM1 is a major regulator of G1/S and G2/M phases of the cell cycle as well as mitotic progression, and can act synergistically with other transcription factors to achieve maximum expression of genes in the G2 phase [18].

In this study, we found that high expression of HMGA1 and FOXM1 is a common molecular event in a variety of cancers, and they have common effects on cell cycle. Furthermore, we explored the expression and effects of HMGA1 and FOXM1 in cancer, and their regulatory mechanism of cell cycle. Our results provide a deep understanding of the biological function of HMGA1 and FOXM1 in cancer cell proliferation.

## 2. Materials and Methods

### 2.1. Datasets and Data Preprocessing

Using The Cancer Genome Atlas (TCGA, https://portal.gdc.cancer.gov/, accessed on 3 July 2022) database, we analyzed the expression of HMGA1 and FOXM1 in tumor tissue and normal tissue. Pan-cancer mRNA expression data were downloaded from the University of California Public Database (UCSC) Xena website (https://xenabrowser.net/datapages, accessed on 3 July 2022). The survival *p* values of corresponding tumor tissues were retrieved from the Gene Expression Profile Interaction Analysis (GEPIA) website (http://gepia2.cancer-pku.cn/, accessed on 13 May 2023) and the correlation between the HMGA1 and FOXM1 in solid tumors was analyzed by Pearson correlation analysis [19]. ROC curves (Receiver Operating Characteristic Curve) were analyzed using “survival” and “timeROC” R packages, and clinicopathological correlation heat maps were analyzed using “limma” and “ComplexHeatmap” R packages. The relevant clinicopathological features were analyzed using “ggpubr” and “limma” R packages. The immunohistochemical images were obtained from the Human Pathology Atlas project (HPA) (https://www.proteinatlas.org). On the premise that false discovery rate (FDR) < 0.05 and |log2(Fold change)| > 2, we applied the “limma” package to screen differentially expressed genes. To further explore the functional roles of HMGA1 and FOXM1 in cancer, we used the “VennDiagram” R package (v 1.7.3) to collect the common target genes of HMGA1 and FOXM1.The “ggplot2”, “clusterProfiler”, “org.Hs.eg.db” and “enrichplot” R package (v 3.3.3) were used for Gene Set Enrichment Analysis (GSEA) and Kyoto Encyclopedia of Genes and Genomes (KEGG) to demonstrate significant functions and pathways between the two groups.

### 2.2. Cell Culture and Treatments

Human non-small cell lung cancer cell line H1299 was obtained from the Cell Repository, Chinese Academy of Sciences (Shanghai, China) and maintained in HEPES-containing RPMI-1640 medium (HyClone, Logan, UT, USA) supplemented with 10% fetal bovine serum (FBS, PAN Seratech, Aidenbach, Germany) and 1% penicillin/streptomycin (Beyotime, Nantong, China) in a humidified incubator with 5% CO_2_ at 37 °C.

### 2.3. Cell Cycle

H1299 cells were subjected to siRNA interference and collected for cell cycle analysis. In brief, cells were stained using the Cell Cycle Kit (MultiSciences, Hangzhou, China) according to the manufacturer’s instructions. The cell cycle distribution was examined by flow cytometry (CytoFLEX S, Beckman, Brea, CA, USA). 

### 2.4. The siRNAs and Knockdown Experiments

H1299 cells were plated in 12-well plates, 100,000/well, and cultured to 70–80% for small RNA interference. The siRNAs of HMGA1 and FOXM1 were referenced to previous publications synthesized by Sangon Biotech (Shanghai, China). For knockdown, the Lipofectamine^®^ RNAiMAX Transfection Reagent (Invitrogen, Darmstadt, Germany) was used following the manufacturer’s instructions. Cells were collected 72 h post siRNA transfection. 

### 2.5. Western Blot, Co-Immunoprecipitation and Immunofluorescence Staining 

Total proteins were extracted with RIPA quick cracking liquid supplied with 1%PMSF (Solarbio, Beijing, China). The proteins were separated by 12% SDS- PAGE. The primary antibodies used in this study were HMGA1 (#39615, Active Motif, Carlsbad, CA, USA), FOXM1(#5436, Cell Signaling Technology, Danvers, MA, USA), ACTIN (#AC006, ABclonal, Woburn, MA, USA), CCNB1(#5436, Cell Signaling Technology) and PLK1(#sc-17783, Santa Cruz, CA, USA). The secondary antibody bound to peroxidase was purchased from ImmuoResearch company, West Grove, PA, USA. The signal was visualized using the chemilluminescence reagent of Western Bright ECL (Advansta, San Jose, CA, USA).

For the co-IP experiment, cells were lysed with cell X-Lysis buffer (Cell Signaling Technology, USA) for 10 min to extract protein. Subsequently, the lysed samples were added to pre-washed protein A/G agarose beads (Santa Cruz Biotechnology, Santa Cruz, CA, USA), then IgG (#A7016, Beyotime), FOXM1 and HMGA1 antibodies were added to the supernatant and rotated overnight at 4 °C. After the incubation, the beads were washed and proteins were eluted by boiling the beads for 10 min in SDS sample buffer for Western blot analysis.

For immunofluorescence staining, cells were cultured on glass coverslip, then fixed with 4% paraformaldehyde solution and permeabilized with 0.5% Triton X-100 in PBS, and then blocked with 1% donkey serum albumin (Solarbio, China). The primary antibodies HMGA1 (1:200) and FOXM1(1:100) were incubated overnight at 4 °C. Cells were then washed with PBS and incubated with the secondary antibodies ABflo™ 594-conjugated Goat Anti-Rabbit IgG (H+L) (1:500, AS039, ABclonal) or Alexa Fluor 488 Donkey Anti-Rabbit (1:500, ab150073, Abcam, Cambridge, UK). The nuclei were labeled with DAPI (Solarbio, China). The sections were observed under an inverted confocal laser scanning microscope (TCS SP8, Leica, Deerfield, IL, USA). 

### 2.6. RNA Extraction and qRT-PCR Analysis

Total RNA was extracted with TRIzol reagent (OMEGA, Washington, DC, USA) following the manufacturer’s instructions. A High-Capacity cDNA Reverse Transcription Kit (CWBio, Beijing, China) was used for reverse transcription. Quantitative RT-PCR was performed on a LightCycler 480 II instrument (Roche, Carlsbad, CA, USA) using a LightCycler 480 SYBR Green I Master. The primers of HMGA1 and FOXM1 were used as in our previous papers [20,21]. Primers for CCNB1, PLK1, TTK, KIF20A, BUB1, and ACTIN were purchased from Sangon Biotech Company (Shanghai, China).

### 2.7. Statistical Analysis 

GraphPad Prism 8.3 software was used for statistical testing, and *p* < 0.05 was considered statistically significant. Data are presented as mean ± standard deviation (SD), and at least three independent individuals or replicates were used per treatment. Using Pearson’s correlation coefficient to evaluate the relationship between two variables. Kaplan–Meier and log-rank tests were used for survival analysis. One-way analysis of variance (ANOVA) was used to compare the differences among groups, followed by pairwise analysis, and results were adjusted for Bonferroni correction.

## 3. Results

### 3.1. HMGA1 and FOXM1 Are Up-Regulated in Pan-Carcinomas and Have Prognostic Value in LUAD, LIHC and PAAD

**Figure 1:**
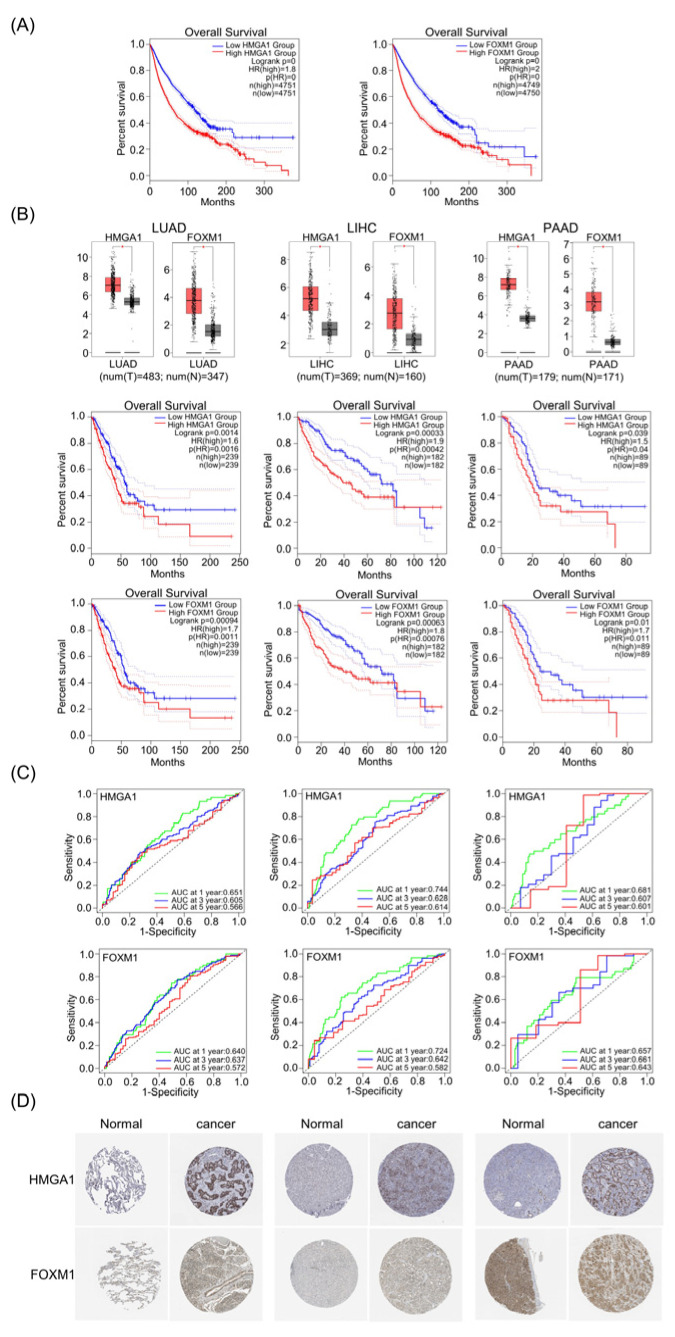
HMGA1 and FOXM1 were overexpressed and negatively correlated with prognosis in LIHC, LUAD and PAAD. (**A**) Overall survival of HMGA1 and FOXM1 in various types of cancer by GEPIA web. (**B**) HMGA1 and FOXM1 expression and overall survival in LUAD, LIHC and PAAD. * indicates *p* < 0.05. (**C**) Time-dependent ROC curves of HMGA1 and FOXM1 predicted the 1-year, 3-year and 5-year survival rates of LUAD, LIHC and PAAD (significance discrimination of AUC: 0.5 < AUC < 0.6 = poor discrimination, 0.6 < AUC < 0.7 = moderate discrimination, 0.7 < AUC < 0.8 = acceptable discrimination, 0.8 < AUC < 1 = excellent discrimination). (**D**) The immunohistochemical images were obtained from The Human Pathology Atlas project (HPA), showing the trend of differential expression of LUAD and LIHC in normal tissues and cancer tissues.

Utilizing the TCGA database, mRNA expression levels of HMGA1 and FOXM1 were compared between various cancers and the corresponding normal tissues. As showed in Figure S1A, either HMGA1 or FOXM1 expression significantly increased in most types of cancer. Moreover, high expression of HMGA1 and FOXM1 was associated with a poor prognosis in the overall cancers (Figure 1A). To give a further demonstration, we collected cancers by setting the criteria that both HMGA1 and FOXM1 were no less than two-fold higher and the correlation coefficient was greater than or equal to 0.5. We found that HMGA1 and FOXM1 were associated with poor prognosis for three cancers with high morbidity and mortality, namely lung adenocarcinoma (LUAD), liver hepatocellular carcinoma (LIHC) and pancreatic adenocarcinoma (PAAD) (Figure 1B, Table S1). Furthermore, HMGA1 and FOXM1 were also evaluated with time-dependent ROC analysis in the LUAD, LIHC and PAAD for their predictive potential benefits. The areas under the ROC curve (AUC) of the prognostic model showed that HMGA1 and FOXM1 had moderate discrimination performance in lung adenocarcinoma patients with 1- and 3-year OS (overall survival), and poor discrimination in 5-year OS. Meanwhile, the time-dependent ROC curve of HMGA1 and FOXM1 for OS in LIHC showed an acceptable discrimination at 1-year OS and moderate discrimination at 3-year OS. At 5-year OS, the ROC analysis provided moderate discrimination for the HMGA1 and poor discrimination for the FOXM1 in LIHC, respectively. In PAAD, both HMGA1 and FOXM1 had moderate discriminative predictive power at 1-, 3-, and 5-year OS (Figure 1C). These data revealed that HMGA1 and FOXM1 had good predictive power in the three cancers of LUAD, LIHC and PAAD.

### 3.2. HMGA1 and FOXM1 Are Associated with the Prognostic Features of LUAD, LIHC and PAAD

We further analyzed and compared the protein level of HMGA1 and FOXM1 in cancer tissues and normal ones based on the immunohistochemical staining. As displayed in Figure 1D, an increased protein level of HMGA1 and FOXM1 in LUAD and LIHC cancer tissues was detected compared to normal ones. However, there was only one case of immunohistochemistry of normal pancreatic tissue in the HPA database and the FOXM1 expression level was high, so there was no significant FOXM1 high expression in PAAD. Then, we investigated the relationship between the prognostic features and clinicopathological features of HMGA1 and FOXM1 (Figures S1B and S2). We found that the risk features of HMGA1 and FOXM1 in LUAD were closely related to patients’ gender, tumor stage and T classification, while HMGA1 was also correlated with N classification (Figure S2A, *p* < 0.05). Comparatively, the risk characteristics of HMGA1 and FOXM1 in LIHC were correlated with patients’ grade, tumor stage and T classification (Figure S2B, *p* < 0.05). Unexpectedly, FOXM1 was only associated with grade in PAAD (*p* < 0.05), and there was no association between the HMGA1 risk signature and clinical features (Figure S2C). These above results suggest that HMGA1 and FOXM1 have a good prognostic value for LUAD, LIHC and PAAD patients.

### 3.3. HMGA1 and FOXM1 Are Positively Correlated in Expression and Have Common Effects on Cell Cycle

**Figure 2:**
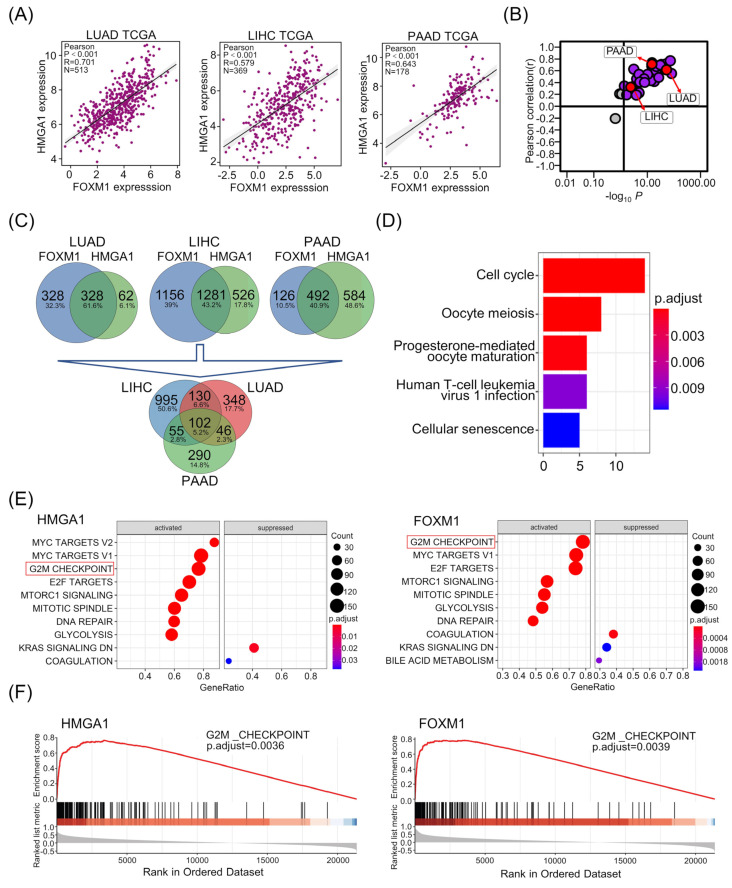
HMGA1 and FOXM1 were positively correlated in expression and had common effects on cell cycle. (**A**) Pearson correlation analysis of expression of HMGA1 and FOXM1 in LUAD, LIHC and PAAD from TCGA Database. The expression level change was indicated as log2 (TPM+0.001). (**B**) Position of LUAD, LIHC and PAAD within the Pearson correlation analysis. (**C**) The flow diagram shows intersection of differentially expressed genes in the three different cancers and the number of genes in each intersection. (**D**) KEGG analysis on biological processes of the 102 overlapped genes. The horizontal axis represents the number of genes. (**E**) The most fifteen significantly enriched KEGG pathways based on overlapping genes in HMGA1 and FOXM1 overexpression in LUAD from TCGA data. (**F**) Enrichment of genes in the representative pathways by GSEA function analysis.

We conducted a comprehensive correlation analysis of HMGA1 and FOXM1 to explore the potential relationship between HMGA1 and FOXM1. Pearson correlation analysis displayed that expression of HMGA1 was positively correlated with that of FOXM1 in all analyzed cancer samples in TCGA databases from GEPIA (r = 0.67, *p* = 0, Figure S2D). The corresponding significance is demonstrated as LUAD (r = 0.701, *p* < 0.001, n = 513), LIHC (r = 0.579, *p* < 0.001, n = 369) and PAAD (r = 0.643, *p* < 0.001, n = 178) (Figure 2A). Moreover, the Pearson correlations of LUAD, LIHC and PAAD among all types of cancer are displayed in Figure 2B.

To further determine their biological effects in cancers, we first collected all significantly changed genes in HMGA1- or FOXM1-overexpressed samples, and made the intersection within the three cancers LUAD, LIHC and PAAD. A total of 625, 1281 and 492 genes were collected in LUAD, LIHC and PAAD, respectively (|log_2_FC| > 1, all *p* < 0.05). After the removal of non-coding RNA, the intersection was conducted and 102 common genes were collected among the three cancers (Figure 2C, Table S2). Finally, we performed the biological classifications of these common genes. The top five enrichment biological roles of cell cycle, oocyte meiosis, progesterone-mediated oocyte maturation, human T-cell leukemia virus 1 infection and cellular senescence were demonstrated based on Kyoto Encyclopedia of Genes and Genomes (KEGG) analysis (Figure 2D). Cell cycle was the most significant related pathway and altered genes are listed in Table 1. Given that HMGA1 and FOXM1 had the highest correlation and the strongest significance, and the most intersection of differential genes in LUAD among the three tumors, we speculate that HMGA1 and FOXM1 are most closely related in LUAD. Subsequently, the biological function of HMGA1 and FOXM1 in LUAD was analyzed using Gene Set Enrichment Analysis (GSEA). The HALLMARK gene set showed that HMGA1 had similar biological functions to FOXM1 and was mainly enriched in the MYC, G2/M and E2F pathways (Figure 2E), and had obvious significance in the G2/M pathway (Figure 2F, *p* < 0.001).

### 3.4. HMGA1 and FOXM1 Cooperatively Regulate G2/M Phase

**Figure 3:**
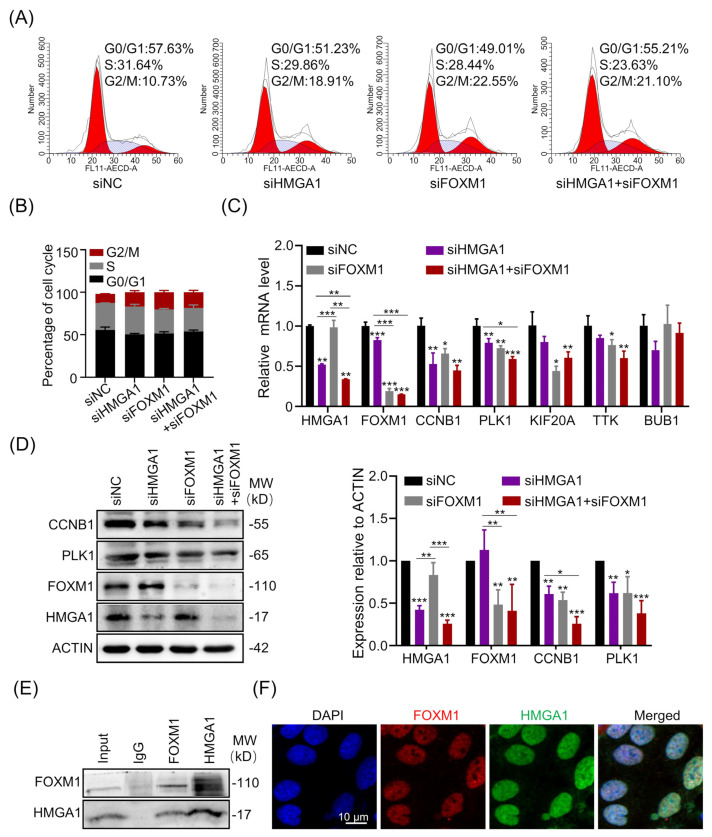
HMGA1 and FOXM1 cooperatively regulate the G2/M phase. (**A**) The cell cycle analysis with flow cytometry after siRNAs mediated knockdown of HMGA1 and FOXM1 in H1299 cells. The percentage of each phase was given based on three independent repeats of experiments. (**B**) Quantitative analysis of each phase of the cell cycle. (**C**) The qRT-PCR validation of mRNA expression of G2/M-phase-related genes HMGA1, FOXM1, PLK1, CCNB1, KIF20A, TTK and BUB1. Values are mean ± SD, n = 3. (**D**) Protein expression levels of HMGA1, FOXM1, PLK1 and CCNB1 after HMGA1 and FOXM1 knockdown. Western Blot band signals were quantified and each value was derived from three independent replicate experiments. (**E**) The complex formation between HMGA1 and FOXM1 protein based on co-immunoprecipitation. (**F**) Representative immunofluorescence images of FOXM1 and HMGA1 localization using laser scanning confocal microscope. * indicates *p* < 0.05; ** indicates *p* < 0.01; *** indicates *p* < 0.001.

In order to demonstrate the functional regulation on cell cycle, we chose non-small cell lung adenocarcinoma cell line H1299 for further experimental confirmation. First, we utilized specific siRNAs targeting HMGA1 and FOXM1 with the efficiency verified both at mRNA and protein levels. Then, through cell cycle analysis, a common increased percentage of cells at G2/M phase was observed in siHMGA1 (18.91%), siFOXM1 (22.55%) groups and siHMGA1+siFOXM1 (21.10%) compared with siNC treatment (10.73%), which is consistent with the overlapped genes, most of which are G2/M-phase-related (Figure 2F and Figure 3A,B, Table 1). To verify the possible targets, we selected five key G2/M phase regulatory genes, polo like kinase 1 (PLK1), cyclin B1 (CCNB1), kinesin family member 20A (KIF20A), TTK protein kinase (TTK) and BUB1 mitotic checkpoint serine/threonine kinase (BUB1), for the analysis of their mRNA expression level with knockdown of HMGA1 and FOXM1 (Figure 3C). The mRNA levels of PLK1 and CCNB1 were significantly decreased both in HMGA1 and FOXM1 knockdown cells. KIF20A and TTK had the downregulated expression only in FOXM1 knockdown cells, while BUB1 had no alternation in knockdown cells. In addition, protein levels of PLK1 and CCNB1 were confirmed to be decreased, especially, CCNB1 had a more significant alternation after combined siRNAs treatment (Figure 3D). These results suggested that PLK1 and CCNB1 were the common targets of HMGA1 and FOXM1 in regulating G2/M phase.

Considering the molecular features of HMGA1 acting as the docking site for other transcription factors and FOXM1 in regulating cell cycle, we conducted co-immunoprecipitation to determine whether HMGA1 and FOXM1 had an interaction. As shown in Figure 3E, FOXM1 or HMGA1 were identified as the components of the immunoprecipitated solution using HMGA1 or FOXM1 antibodies, respectively. To give a further verification, immunofluorescence staining was conducted, and the colocalization of HMGA1 and FOXM1 was observed within the cellular nucleus (Figure 3F). These results strongly indicated the cooperative regulatory role of HMGA1 and FOXM1.

## 4. Discussion

Previous studies reported that transcription factors performed their function in two different manners. The direct model involves transcription factors binding directly to consensus DNA sequences in cis-regulatory regions, called enhancers. The indirect model involves protein complexes composed of different cofactors that cluster at DNA target sites and assist in the recruitment of transcription factors to increase regulatory flexibility [22,23]. HMGA1 is a structural transcription factor of chromatin. It does not act directly as a transcription factor, but rather coordinates the assembly and binding of other transcription factors on DNA and regulates the transcription of many important oncogenes [24]. The main manner of its oncogenic mechanism is that HMGA1 stabilizes the binding of transcription factors to DNA by changing chromatin structure, thereby initiating the transcription of downstream genes. Meanwhile, HMGA1 increases the affinity of other transcription factors enriched on DNA for their respective recognition sequences, enhances protein-protein synergism among other transcription factors and promotes the expression of genes associated with tumor progression and metastasis [25,26,27]. For example, HMGA1 enhances binding to NF-KB and ATF-2 and promotes synergistic interaction between NF-KB and ATF-2 by inducing local DNA conformational changes [28]. In this study, we found that HMGA1 was overexpressed and predicted poor clinical outcomes in most types of cancers. Therefore, a deeper understanding of the molecular mechanism underlying HMGA1 will be crucial for the discovery of new targets for effective therapies.

As a member of the forkhead family of transcription factors, accumulating evidence suggests that FOXM1 is involved in tumorigenesis by activating specific transcriptional pathways [29]. As a master regulator of the cell cycle, FOXM1 can be directly phosphorylated and activated to regulate G2/M transition and normal mitotic progression by forming a complex with the mitotic kinase PLK1 [30]. In addition, FOXM1 can be upregulated by circRNA circTP63, which promotes cell cycle transition from G1/S phase to G2/M phase, thereby accelerating the proliferation of lung squamous cell carcinoma [31]. Similar to HMGA1, FOXM1 is overexpressed in most tumors and predicts poor prognosis [32]. Importantly, both HMGA1 and FOXM1 exert regulatory activity in the direct form of protein binding to DNA and protein–protein interaction after assisted recruitment. Further exploration the regulatory mechanism between HMGA1 and FOXM1 is necessary and would help identify patients who require aggressive and individualized treatment to improve clinical outcomes.

Using the TCGA dataset, HMGA1 expression was identified to have a positive correlation with FOXM1, especially in LUAD, LIHC and PAAD, suggesting their common importance in cancer. After the intersection of significantly altered genes in the HMGA1 and FOXM1 overexpressed samples, cell cycle was found to be the most enriched term based on KEGG and GSEA analysis, which was in good agreement with previous publications related to the function of both genes [11,31,33]. Further analysis indicated that those altered cell cycle genes were related to G2/M phase, which was also identified by the increased percentage of G2/M phase in HMGA1 and FOXM1 knockdown cells. Furthermore, PLK1 and CCNB1, two key G2/M-regulating genes, were discovered to be the downstream targets. FOXM1 acted as an important regulator of G2/M cell cycle progression in multiple cancers, and interacted with other proteins to synergistically promote cell cycle progression [34]. As a novel molecular partner of HMGA1, the interaction between FOXM1 and HMGA1 increased the transcriptional activity of common target genes to a certain extent [35]. Our co-immunoprecipitation and immunofluorescence staining experiments identified the protein complex formation within the nucleus, indicating the synergistic effect of HMGA1 and FOXM1. HMGA1 and FOXM1 were characteristic of most malignancies. Clearly, with the increase of HMGA1 and FOXM1 expression levels, the clinical and pathological features of patients showed asymmetric distribution. By manipulating proliferative signals, HMGA1 and FOXM1 have become important molecules involved in cancer development and progression. One possible reason is that the interaction between HMGA1 and FOXM1 maintains the stability of FOXM1 protein, thus protecting FOXM1 from ubiquitination and degradation [36,37]. It is also possible that HMGA1 activates the expression of mitotic spindle assembly checkpoint-related genes, which is an important control system of cell cycle, leading to checkpoint damage and chromosome instability and promoting the progression of cancer [38].

In summary, our results provide a further understanding of HMGA1 in cooperation with FOXM1 within the nucleus to regulate gene transcription in controlling G2/M cell cycle progression, which could provide a potential cancer treatment strategy by targeting both HMGA1 and FOXM1 in cancers. 

## Figures and Tables

**Table 1:** The list of overlapping cell cycle genes and expression level changes (LogFC) based on gene ontology analysis using genes from the intersection among LIHC, LUAD and PAAD.

	LIHC		LUAD		PAAD	
	FOXM1	HMGA1	FOXM1	HMGA1	FOXM1	HMGA1
ORC6	2.257	1.305	1.467	1.164	1.283	1.001
ESPL1	2.182	1.102	2.049	1.617	1.348	1.074
CCNB2	2.617	1.722	1.807	1.479	1.661	1.301
PKMYT1	1.613	1.279	1.516	1.172	1.105	1.187
CDC45	2.18	1.455	1.776	1.46	1.324	1.209
TTK	2.747	1.699	2.025	1.544	1.6	1.139
PLK1	2.756	1.844	1.838	1.548	1.451	1.244
E2F1	2.273	1.276	1.448	1.184	1.21	1.046
CDC25C	2.408	1.473	1.698	1.384	1.576	1.378
CDC20	2.559	1.821	2.016	1.602	1.561	1.257
CDC6	2.546	1.54	2.036	1.75	1.435	1.088
CDK1	2.455	1.542	1.612	1.313	1.393	1.102
BUB1B	2.725	1.667	1.852	1.493	1.29	1.033
BUB1	2.633	1.667	1.817	1.422	1.52	1.194

Abbreviation: ORC6: origin recognition complex subunit 6, ESPL1: extra spindle pole bodies like 1, CCNB2: cyclin B2, PKMYT1: protein kinase membrane associated tyrosine/threonine 1, CDC45: cell division cycle 45, TTK: TTK protein kinase, PLK1: polo like kinase 1, E2F1: E2F transcription factor 1, CDC25C: cell division cycle 25C, CDC20: cell division cycle 20, CDC6: cell division cycle 6, CDK1: cyclin dependent kinase 1, BUB1B: BUB1 mitotic checkpoint serine/threonine kinase B, BUB1: BUB1 mitotic checkpoint serine/threonine kinase.

## Data Availability

The data can be shared upon request.

## Supplementary Materials

The following supporting information can be downloaded at: https://www.mdpi.com/article/10.3390/life13051225/s1, Figure S1: The expression and correlation of HMGA1 and FOXM1 in pan-cancer; Figure S2: The relationship between HMGA1 and FOXM1 in the clinicopathological features of LUAD, LIHC and PAAD; Table S1: The expression of HMGA1 and FOXM1 and survival significance in various types of cancer; Table S2: Co-regulatory genes in LUAD, LIHC and PAAD cancers of HMGA1 and FOXM1 overexpressed mRNA samples.

## Abbreviation

HMGA1: the high mobility group AT-Hook 1; FOXM1: Forkhead box M1; ROC: Receiver Operating Characteristic Curve; TCGA: The Cancer Genome Atlas; AUC: The area under the ROC curve; LUAD: lung adenocarcinoma; LIHC: liver hepatocellular carcinoma; PAAD: pancreatic adenocarcinoma; PLK1: polo like kinase 1; CCNB1: cyclin B1; KIF20A: kinesin family member 20A; TTK: TTK protein kinase; BUB1: BUB1 mitotic checkpoint serine/threonine kinase; GEPIA: the Gene Expression Profile Interaction Analysis; HPA: the Human Pathology Atlas Project; GSEA: Gene Set Enrichment Analysis; KEGG: Kyoto Encyclopedia of Genes and Genomes.
