# Correction: Zheng et al. HMGA1 and FOXM1 Cooperate to Promote G2/M Cell Cycle Progression in Cancer Cells. *Life* 2023, *13*, 1225

**DOI:** 10.3390/life15111694

**Published:** 2025-10-31

**Authors:** Qingfang Zheng, Ziyang Luo, Mingjun Xu, Shazhou Ye, Yuxin Lei, Yang Xi

**Affiliations:** Institute of Biochemistry and Molecular Biology, Basic Medical Sciences, Health Science Center, Ningbo University, Ningbo 315211, China; zhengqf910@163.com (Q.Z.); baronlzyang@163.com (Z.L.); 1911074035@nbu.edu.cn (M.X.); yeshazhou@foxmail.com (S.Y.); 216002284@nbu.edu.cn (Y.L.)

## Error in Figure

In the original publication [1], there was a mistake in the second image of Figure 1A: “Low HMGA1 Group; High HMGA1 Group” as published. The corrected image “Low FOXM1 Group; High FOXM1 Group” appears below. The authors state that the scientific conclusions are unaffected. This correction was approved by the Academic Editor. The original publication has also been updated.

**Figure 1:**
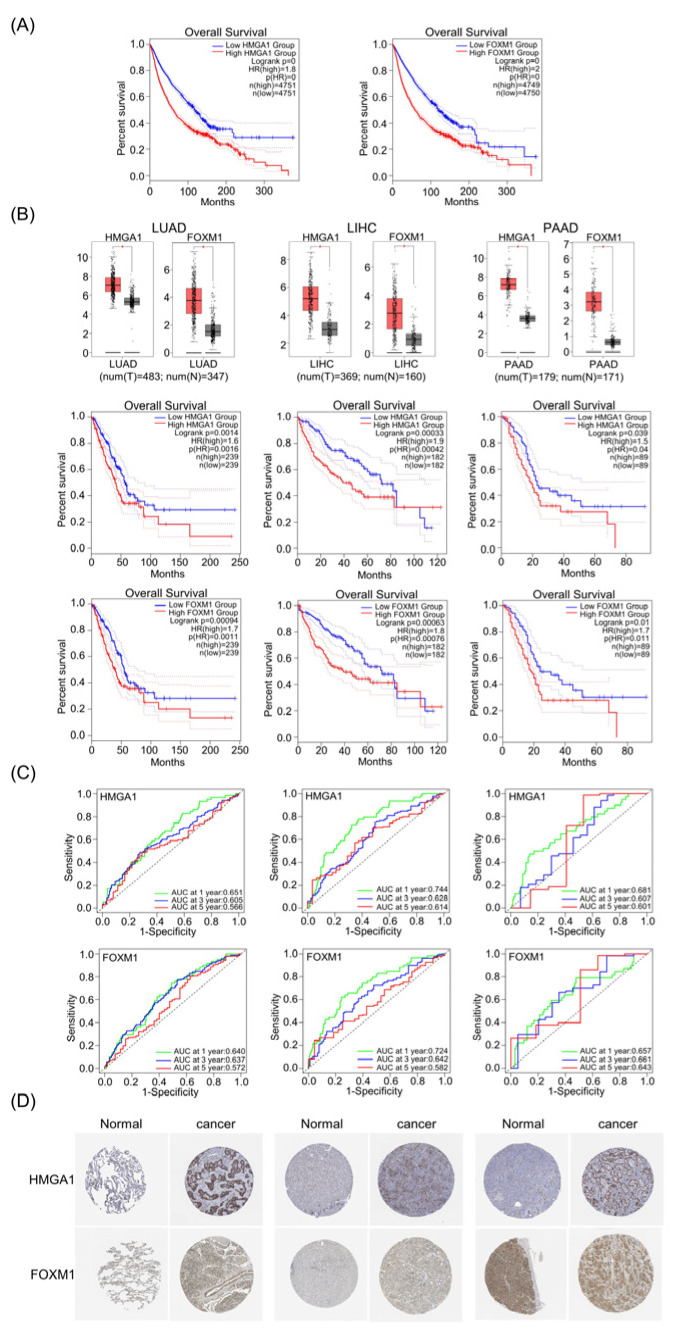
HMGA1 and FOXM1 were overexpressed and negatively correlated with prognosis in LIHC, LUAD and PAAD. (**A**) Overall survival of HMGA1 and FOXM1 in various types of cancer by GEPIA web. (**B**) HMGA1 and FOXM1 expression and overall survival in LUAD, LIHC and PAAD. * indicates *p* < 0.05. (**C**) Time-dependent ROC curves of HMGA1 and FOXM1 predicted the 1-year, 3-year and 5-year survival rates of LUAD, LIHC and PAAD (significance discrimination of AUC: 0.5 < AUC < 0.6 = poor discrimination, 0.6 < AUC < 0.7 = moderate discrimination, 0.7 < AUC < 0.8 = acceptable discrimination, 0.8 < AUC < 1 = excellent discrimination). (**D**) The immunohistochemical images were obtained from The Human Pathology Atlas project (HPA), showing the trend of differential expression of LUAD and LIHC in normal tissues and cancer tissues.
