# Supplementary figures and images for: HMGA1 and FOXM1 Cooperate to Promote G2/M Cell Cycle Progression in Cancer Cells
Source: Life (Basel). 2023 May 22;13(5):1225. doi: 10.3390/life13051225 (PMC10223534; doi:10.3390/life13051225)

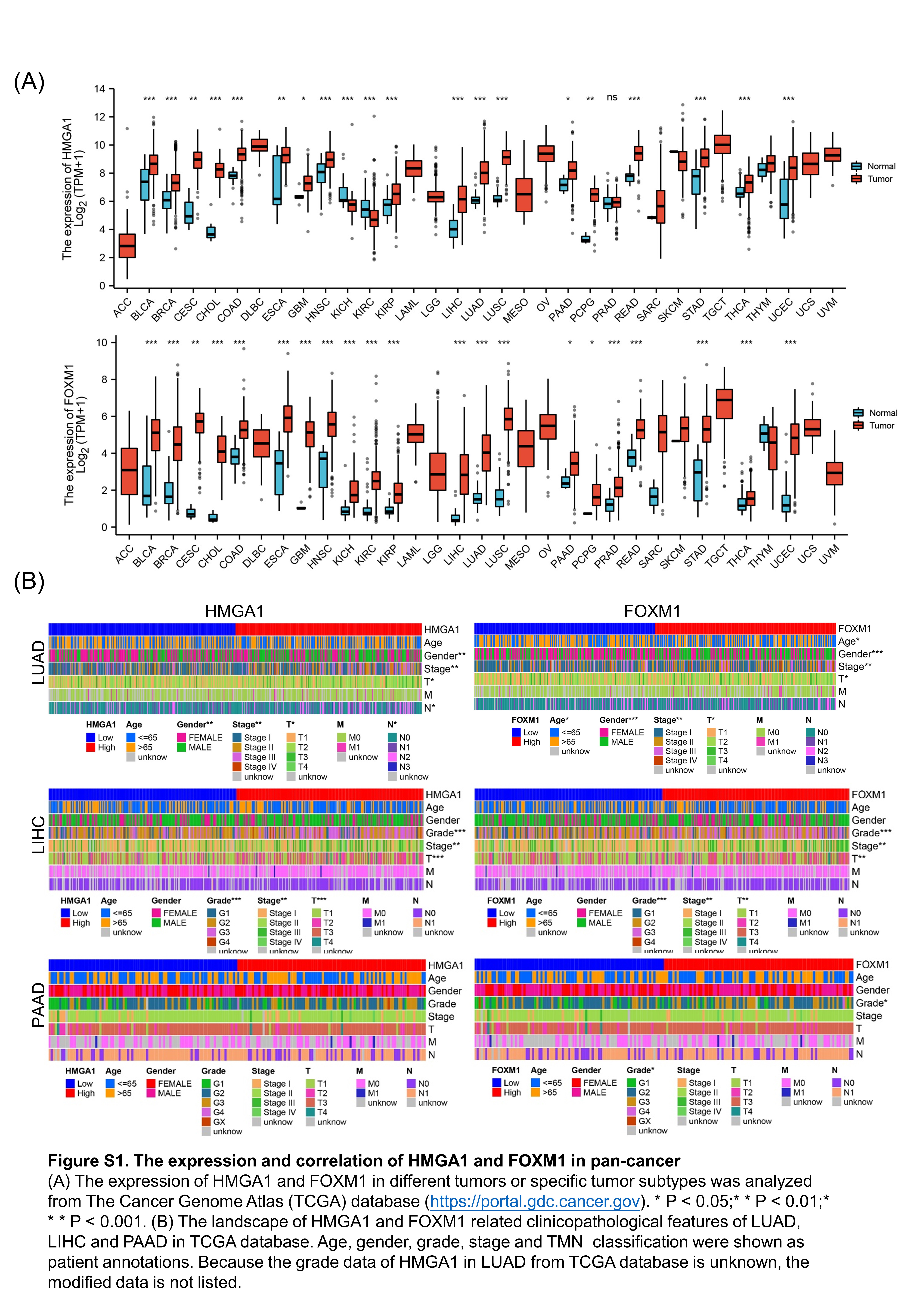

Supplement: Supplementary file 1 [file life-13-01225-s001.zip › Figure S1.jpg]

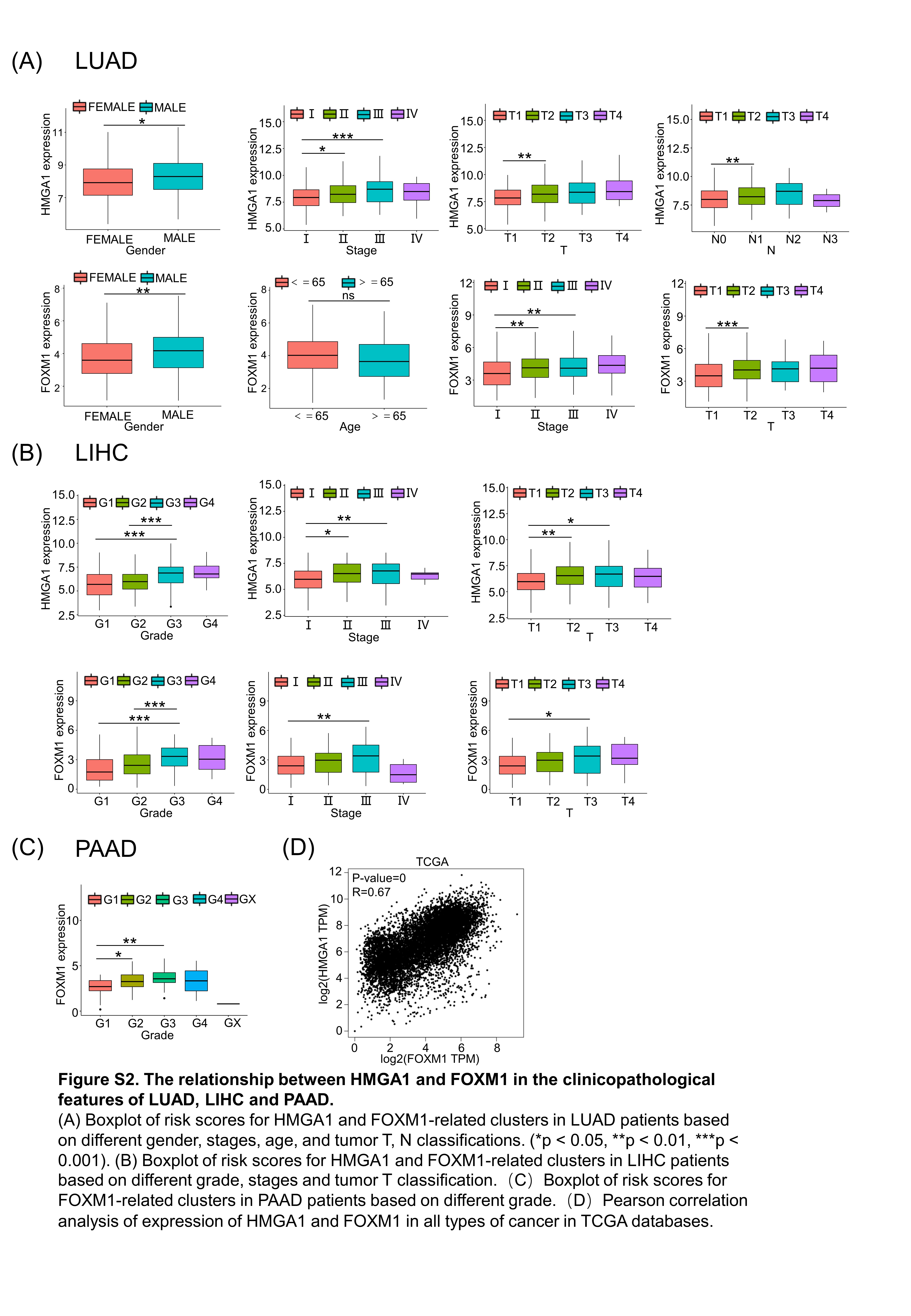

Supplement: Supplementary file 1 [file life-13-01225-s001.zip › Figure S2.jpg]

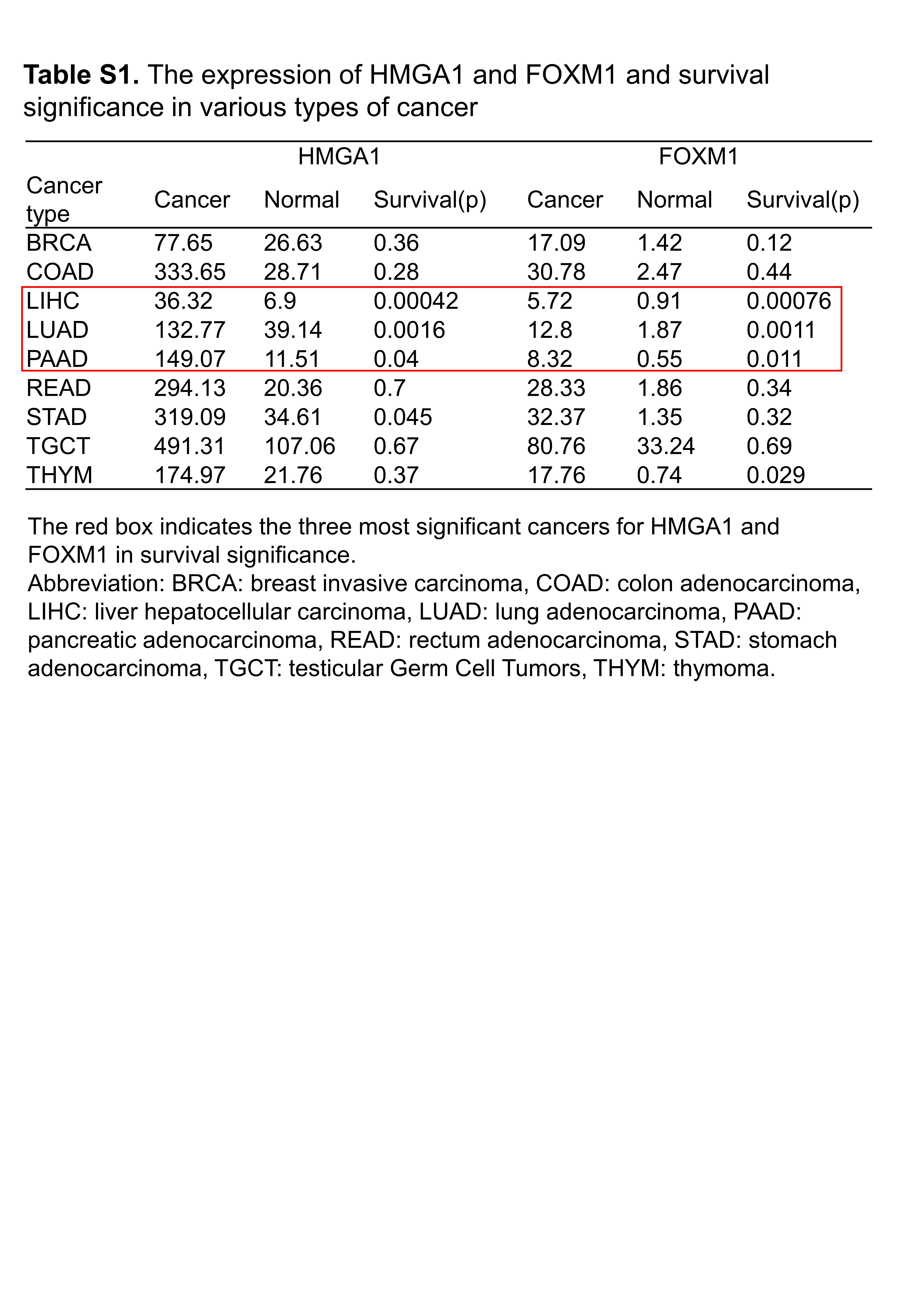

Supplement: Supplementary file 1 [file life-13-01225-s001.zip › Table S1.jpg]

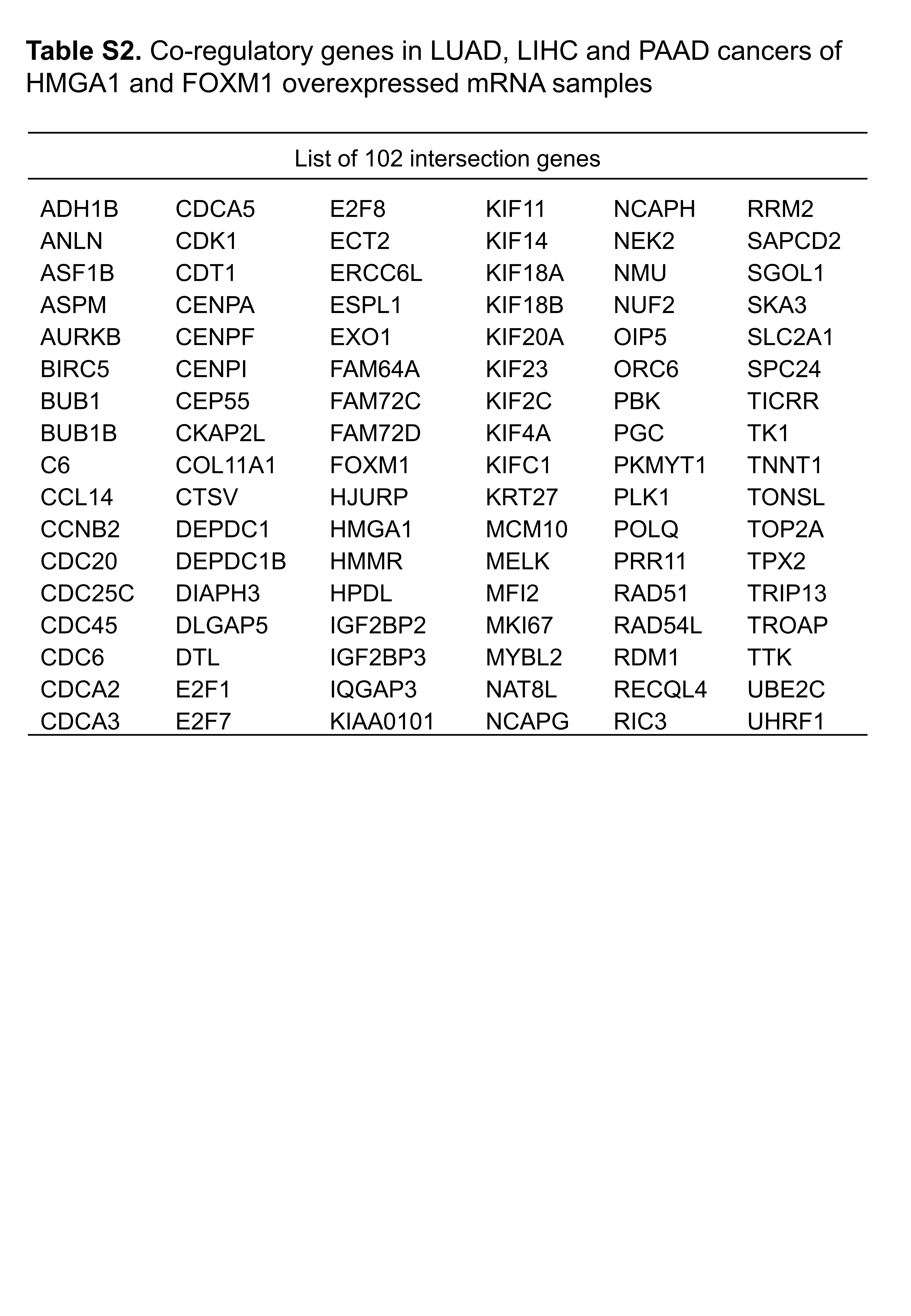

Supplement: Supplementary file 1 [file life-13-01225-s001.zip › Table S2.jpg]
